# Impact of Hysterectomy on Quality of Life, Urinary Incontinence, Sexual Functions and Urethral Length

**DOI:** 10.3390/jcm10163608

**Published:** 2021-08-16

**Authors:** Katarzyna Skorupska, Sara Wawrysiuk, Michal Bogusiewicz, Pawel Miotła, Izabela Winkler, Agnieszka Kwiatkowska, Tomasz Rechberger

**Affiliations:** 1 2nd Department of Gynecology, Medical University of Lublin, ul. Jaczewskiego 8, 20-954 Lublin, Poland; sara.wawrysiuk@gmail.com (S.W.); mbogusiewicz@yahoo.com (M.B.); pmiotla@wp.pl (P.M.); rechbergt@yahoo.com (T.R.); 2 2nd Clinic of Gynecology, St. John’s of Dukla Cancer Center Lublin, 20-090 Lublin, Poland; ikochans@interia.pl; 3 Medical University of Lublin, ul. Jaczewskiego 8, 20-954 Lublin, Poland; gakwiatkowska2604@gmail.com

**Keywords:** hysterectomy, quality of life, urethral length, sexual function

## Abstract

The aim of the study was to evaluate the influence of different types of hysterectomy on UI symptoms, quality of life and sexual functions using dedicated questionnaires. We investigated a correlation between the urethral length (UL), UI symptoms and the length of the cervix (left after LSH and SH) with sexual functions. The study enrolled 500 consecutive women referred for hysterectomy: 121 underwent VH, 171 underwent LSH, 96 underwent SH, 68 underwent TAH, and 44 underwent TLH. The patients filled in the UI-specific questionnaires and FSFI before and 12 months after hysterectomy. The UL was measured by introital ultrasound before and 12 months after hysterectomy. Before surgery, 137 out of 399 (34.3%) patients had UI symptoms; afterwards, 139 (34.8%) indicated the same (*p* > 0.05). There was no statistically significant difference in the UL in the patients before and after the procedure, and the cervix length did not differ between patients after LSH and SH. When the entire investigated population was analyzed, a significant improvement of the QoL was found on the IIQ-7. Hysterectomy performed due to benign diseases has effects on UI regardless of the surgical technique used. The UI symptoms improved only in the patients after LSH. The UL measured 12 months after hysterectomy did not change.

## 1. Introduction

Performing a hysterectomy can cause injuries to several anatomical structures, including the urinary tract, digestive tract and nervous structures, which result in many complications. It is also a risk factor for anxiety, social dysfunction and depression, as the women after the procedure become infertile [1]. Hence, hysterectomy is considered to be a risk factor for urinary incontinence (UI), pelvic organ prolapse (POP) or sexual dysfunction [2]. However, the extent of the hysterectomy and the surgical approach may strongly influence the incidence and severity of symptoms. UI is a symptom characterized by a complaint of involuntary urination [3]. The prevalence of UI in females reaches up to 53.4%, depending on the study population [4]. UI has a severe effect on daily life, affecting the social, familiar and sexual domains [5]. Hysterectomy is a proven risk factor for postoperative UI. In a cohort study conducted by Milsom et al., the prevalence of UI in patients with a history of hysterectomy reached 20.8%, compared to 16.4% in a control group [6]. The exact pathomechanism responsible for the increased risk of UI development after hysterectomy is not fully understood. The loss of anatomical support tissue of the urethra and the bladder, the impairment of the sphincter mechanism and the formation of scar tissue are some of the considered contributing factors [7]. One proposed factor is the urethral length. Kupec et al. proved a significant difference in the UL of patients with stress urinary incontinence (SUI) and overactive bladder syndrome (OAB) versus a control group. Furthermore, the UL was significantly greater (*p* < 0.05) in women with UI [8]. However, these results were not supported in the earlier cross-sectional study by Rostaminia et al., where no significant difference in UL was found between continent and incontinent patients [9].

The data on the influence of hysterectomy on quality of sexual life are contradictory. Some authors claim that the removal of the uterus improves sexual life [10,11], while others state that it deteriorates afterwards [12,13]. Patients after various kinds of hysterectomy claim different results. There are studies which show that sexual life after supracervical hysterectomy can be better than after total hysterectomy [14,15,16], and those which show that the type of surgery does not affect the QoL afterwards [10,17]. 

The data shows that the QoL after hysterectomy is compromised by the fact that women are infertile after the procedure and should be educated about possible fertility preservation techniques [18,19]. Few clinicians would dispute the high level of distress in women affected by infertility.

The purpose of the study was to evaluate the influence of different types of hysterectomy—vaginal (VH), abdominal (TAH), laparoscopic (TLH), supracervical abdominal (SH) and supracervical laparoscopic (LSH)—on UI manifestation, QoL and sexual functions using dedicated questionnaires. Moreover, we also investigated whether there is a correlation between the urethral length and UI symptoms, as well as the length of the cervix (left after LSH and SH) on sexual functions. 

## 2. Materials and Methods

The protocol of the study was carried out in accordance with the European Communities Council Directive of 22 September 2010 (2010/63/EU) and various acts of Polish legislation. The Local Ethics Committee approved the study (KE-0254/69/2018). Before inclusion, all of the patients gave written informed consent for participation in the study. The study group consisted of 500 (otherwise healthy) consecutive women referred to the Gynecology Department for hysterectomy due to benign indications (Table 1). These patients included 121 who underwent vaginal hysterectomy (VH), 171 who had laparoscopic supracervical hysterectomy (LSH), 96 treated by means of abdominal supracervical hysterectomy (SH), 68 who underwent total abdominal hysterectomy (TAH), and 44 who had total laparoscopic hysterectomy (TLH). We established the sample size based on Comfrey and Lee’s suggestion that 50 is very poor, 100 is poor, 200 is fair, 300 is good, 500 is very good, and 1000 or more provides a perfect sample size [20].

The patients filled out the Urogenital Distress Inventory-6 (UDI-6), Incontinence Impact Questionnaire-7 (IIQ-7), International Consultation on Incontinence Questionnaire (ICIQ) and Female Sexual Function Index (FSFI) before and 12 months after hysterectomy. Pelvic Organ Prolapse (POP) was assessed using the Pelvic Organ Prolapse Quantification (POP-Q) system [21].

The UL was measured by introital ultrasound before and 12 months after hysterectomy. The measurements were taken in a standardized manner (the bladder filled to 200–400 mL). The length of the cervix was measured in patients who underwent LSH and SH 12 months after the procedure. The main inclusion criteria were: fibroids, abnormal uterine bleeding and pelcic organ prolapse. The exclusion criteria were as follows: malignancy, lack of consent, reduced capability of understanding the survey, any comorbidities which may influence the general well-being after surgery, bladder filling less than 200 mL or more than 400 mL, previous history of pelvic floor surgery, and a lack of consent. POP was the most common indication for hysterectomy in the VH group of patients. We included only the patients who did not undergo any surgery in the meantime between the surgery and follow up. All of the tests were carried out in agreement with the International Continence Society standards.

### 2.1. Questionnaires

The ICIQ-SF assesses the frequency, intensity and influence of UI on QoL in research and clinical practice. The ICI recommends ICIQ-SF (validation level-A). Moreover, the ICIQ-SF’s validity, reliability and responsiveness to change has been confirmed [22]. The questionnaire consists of 4 items: Frequency of UI, Amount of leakage, Overall impact of UI, and a Self-diagnostic item. The occurrence or absence of symptoms and their intensification in the ICIQ-SF questions is measured in 5-point Likert scales. The total score is from 0 to 21. Greater values indicate increased symptom severity. 

UDI-6 is a condition-specific QoL instrument. The ICI assessed UDI-6 as validation level A [22]. It consists of six items: 1—Frequent urination, 2—Leakage related to feeling of urgency, 3—Leakage related to activity, 4—Coughing or sneezing with small amounts of leakage (drops), 5—Difficulty emptying the bladder, and 6—Pain or discomfort in the lower abdominal or genital area. Greater values in the UDI-6 indicate higher disability. The overall score is from 0 to 100 [23].

The IIQ-7 is a UI-specific psychometric questionnaire. The psychosocial influence of UI is assessed by this questionnaire. The ICI validated the IIQ-7 as A [13]. It consists of 7 items: 1—Household chores, 2—Physical recreation, 3—Entertainment activities, 4—Travel >30 min away from home, 5—Social activities, 6—Emotional health (nervousness, depression, etc.), and 7—Feeling frustrated; which is subdivided into 4 domains: PA—physical activity (items 1 and 2), TR—travel (items 3 and 4), SA—social activities (item 5), and EH—emotional health (items 6 and 7). The overall score is from 0 to 100 [24].

The FSFI questionnaire measures sexual function. It consists of 19 questions which enable an estimation of sexual function over the four weeks prior to the assessment. The subscale scores ranged from 0 to 6. Higher scores indicate better sexual function. Sexual dysfunction is found in patients who obtained 27.50 or less points in PL-FSFI [25].

The UDI-6, IIQ-7, ICIQ and FSFI have been successfully translated into and validated in Polish [25,26].

### 2.2. Statistical Analysis

The statistical analysis was performed with Statistica.StatSoft 13.0 (StatSoft, Tulusa, OK, USA). We collected the clinical data prospectively in a customized database and analyzed them retrospectively. The Shapiro-Wilk test and Lilliefors test were used to verify the normality within the groups. Student’s T test for independent samples, Student’s T test for dependent samples and the Wilcoxon signed-rank test for dependent samples were used to verify the statistical hypotheses as appropriate. The correlation between the results of UDI-6, ICIQ, UL and cervix length were compared using Spearman’s correlation coefficient. The demographic and clinical data are shown as frequencies and percentages. The continuous variables are expressed as the mean ± standard deviation, or the median. A value of *p* < 0.05 was considered statistically significant.

## 3. Results

Of the 500 women who underwent hysterectomy, 399 (79.8%) came for a follow up. They underwent clinical examination, completed all of the questionnaires, and had their urethra (and cervix) measured. The mean observation time was 12.1 months. The demographic characteristics of the study group patients are listed in Table 1. The mean age differed among the groups, with the oldest patients constituting the VH group. Moreover, the mean BMI was lower in the patients who underwent laparoscopy (the LSH and SH groups) in comparison with the TAH patients. BMI did not change significantly between the hysterectomy and follow up (Table 1).

We investigated BMI, parity, age, the type of hysterectomy and preoperative UI, and their impact on postoperative UI occurrence. The regression model did not show any independent factor of UI in the investigated population.

Before the surgery, 137 out of 399 (34.3%) patients had UI symptoms—43 (28.6%) from the LSH group, 14 (29.7%) from the TAH group, 5 (17.8%) from the TLH group, 53 (51.4%) from the VH group and 22 (30.9%) from the SH group. After the surgery, 139 women (34.8%) declared UI symptoms—43 (28.6%) from the LSH group, 28 (59.5%) from the TAH group, 11 (39.2%) from the TLH group, 40 (38.8%) from the VH group and 17 (23.9%) from the SH group. The differences were not statistically significant.

When the entire investigated population was analyzed, a significant improvement of the QoL was found on the IIQ-7. However, when the groups were analyzed separately, the QoL calculated from the IIQ-7 improved only in patients after LSH. According to the ICIQ, patients after SH had significantly more UI symptoms postoperatively. This finding was not observed in the other groups. The results of the UDI-6 questionnaire did not show any change in UI or QoL. 

There were no statistically significant differences in the UL in the patients from the study groups before and after the procedure, and in all of the study groups, the UL did not change after surgery (Table 2). There was no correlation between the UL and questionnaire scores.

We observed no change in sexual function 12 months after hysterectomy in any of the study groups. Here, only the results of the 297 (74.4%) patients who were sexually active before and after the surgery were taken into account. The cervix length did not differ between the patients after LSH (3.58 cm) and SH (3.53 cm) 12 months after the procedure. There was no statistically significant correlation between the cervix length and the questionnaire results.

Table 2 shows the mean +/−SD ICIQ, UDI-6, IIQ-7 and FSFI questionnaire scores among the study group patients, whereas Table 3 shows the urethral length before and after hysterectomy.

## 4. Discussion

Hysterectomy is considered a risk factor for postoperative UI, regardless of the type of procedure [2]. In our study, we investigated the occurrence of UI symptoms after various types of hysterectomy and their impact on quality of life. Our results show that all of the patients claimed a decreased severity of UI symptoms and improved QoL regardless of the procedure used, as evaluated by the IIQ-7. Specifically, only supracervical hysterectomy appeared to have a certain impact on the occurrence of UI. According to their IIQ-7 scores, patients after LSH had significantly fewer UI symptoms. In contrast, patients after SH reported higher severity UI symptoms on the ICIQ after the procedure. We also investigated the change in urethral length between the time point before hysterectomy and afterwards, and we did not observe any change. To our knowledge, this is the first study which evaluated the impact on urethral length via ultrasonography. 

Altman et al., in a cohort study, found no correlation between the incidence of UI and the type of hysterectomy [27]. However, a systematic review by Longo et al. revealed a higher frequency of UI after subtotal hysterectomy compared to total hysterectomy [28]. Moreover, the patient’s satisfaction with the surgery and impact of hysterectomy on UI is strongly connected to other factors, such as the number of vaginal deliveries, being overweight, and daily urge symptoms without incontinence prior to the operation—which has a negative influence on the rate of remission of UI after hysterectomy [29]. We investigated BMI, parity, age, the type of hysterectomy and preoperative UI, and their impact on postoperative UI occurrence. The regression model did not show any independent factor of UI in the investigated population. Total abdominal hysterectomy (TAH) causes the biggest tissue trauma of all of the abovementioned surgeries. Previous studies suggested that TAH causes alterations in UL and urethral function secondary to the urethra and urinary bladder neurovegetative nerve plexus damage, vascular changes and anatomic changes of the bladder neck position [30]. Furthermore, other data suggest that women with preoperative incompetence of the bladder neck may tend to develop SUI after TAH due to a reduction in their urethral closure pressure [31]. These observations were not supported by our results. Similarly, in a study conducted by Dimitri et al., in which the urinary bladder position and mobility were ultrasonographically evaluated 12 months after TAH, no change in the urethral supportive structures and no increase in the SUI rate were reported [32]. 

As the urethra is the main structure involved in the continence mechanism, its anatomy has been subjected to extensive research. The data were collected during cadaver studies, with measurements using a Foley catheter, during a urodynamic study based on the urethral pressure profile, magnetic resonance imaging (MRI), and ultrasound examination [33]. Transperineal urethral ultrasound is the most common and the safest method of measuring urethral length. It is measured from the bladder neck to the external meatus along the urethral longitudinal axis. The acquisition has been proven to be accurate when using endovaginal ultrasound with 360° rotation, and this shows statistical agreement between different investigators [34]. Despite the progress made on UI research, the understanding of the influence of UL on UI symptoms has not yet been determined. The mean urethral length in the whole study group before the procedure was 3.1 cm. This value corresponds with a result of a cohort study on urethral length conducted by Pomian et al. [33]. We did not observe a statistically significant difference in the urethral and cervical length after the procedure, although correlations between hysterectomy and urinary incontinence were observed.

### Sexual Function

Up to 37% of all of the patients undergoing hysterectomy reported a decline in sexual function [35]. Vaginal length after hysterectomy is one of the factors affecting sexual satisfaction. Laparoscopic techniques have been demonstrated to be more successful then AH in preserving the vaginal length from before the procedure [36]. Studies have shown that the use of a uterine manipulator (UM) during laparoscopy prevents the shortening of the vagina and results in the maintenance of sexual function, with some even suggesting the use of a UM during AH [37]. In our study, we observed a small improvement in sexual functions in patients after LSH, but not after TLH. Those changes were not clinically significant. 

The important issue concerning sexual satisfaction after hysterectomy is the preservation of the cervix. In a study conducted by Berlit et al. comparing sexual functioning after TLH versus LSH, the preservation of the cervix showed no improvement in sexual function after surgery [38]. Furthermore, hysterectomy led to an improvement of sexual functioning regardless of which surgical technique was used. In our study, we also observed no clinically significant change in sexual function for any of the hysterectomy techniques. Moreover, Radosa et al., in a prospective nonrandomized trial that assessed sexual function in 237 patients, on comparing VH, LASH and TLH, found that all of the procedures showed an improvement in postoperative sexuality with no statistically significant difference between the procedures [10]. The improvement can be explained by the elimination of vaginal bleeding, coital pain and contraception-related issues [39].

Patients undergoing VH are usually suffering from an advanced stage of pelvic organ prolapse (POP). POP is one of the factors that negatively influence sexual function and sexual satisfaction—not only due to changes in the anatomy of the patient but also because of psychological factors. In addition, the women who received VH were statistically the oldest group of patients, with a mean age of around 65 years old. Hence, we suspected that the FSFI score in this group would be the lowest. Surprisingly, this group of patients score similarly to other groups, and no change in sexual function was observed after hysterectomy. 

One of the strengths of our study was its novel approach to urethral length in ultrasonography and the use of validated questionnaires. The limitation of the study was the relatively small group of patients undergoing TLH compared to the other groups, as well as the single setting of the study.

## 5. Conclusions

The current evidence suggests that hysterectomy for benign diseases has some effects on sexual function and urinary incontinence irrespective of the surgical technique used. We observed an improvement in UI symptoms only in patients after LSH. The urethral length measured 12 months after the hysterectomy did not change.

During the preoperative decision-making process, patients should be informed that hysterectomy performed due to a benign condition is a risk factor for postoperative UI, although it does not influence sexual function regardless of the type of procedure. According to the existing evidence, LSH should be considered as the safest method of hysterectomy, especially in terms of worsening already existing UI. Future studies comparing the safety profile of different hysterectomies in terms of UI should be conducted.

## Figures and Tables

**Table 1:** Demographic characteristics of the study group patients (continuous variables are presented as the mean ± SD).

Parameter	Study Group (n = 399)				
	VH (n = 103)	LSH (n = 150)	SH (n = 71)	TAH (n = 47)	TLH (n = 28)
Age (years) * (mean ± SD)	64.8 ± 10.4	47.2 ± 4.72	47.5 ± 4.72	57.9 ± 9.87	51.8 ± 7.68
BMI Beginning of the study 12 month follow up (kg/m^2^) ** (mean ± SD)	28.3 ± 4.16 28.1 ± 4.87	26.6 ± 4.61 26.3 ± 4.89	28.9 ± 5.96 28.9 ± 5.67	29.5 ± 7.34 29.4 ± 7.45	27.1 ± 4.99 27.2 ± 5.01
Parity (mean ± SD)	2.66 ± 1.31	2.57 ± 1.35	2.58 ± 1.37	2.51 ± 1.39	2.49 ± 1.41
Postenopausal	N = 84 (81.5%)	N = 23 (15.3%)	N = 8 (11.2%)	N = 31 (65.9%)	N = 12 (42.8%)

* There were statistically significant differences in the mean age in the comparisons among all of the groups (*p* < 0.01), except the one between LSH and SH. ** The mean BMI was statistically significantly higher in the TAH patients when compared to the LSH and TLH groups (*p* < 0.05). There was no statistically significant difference between the BMIs at the beginning and at the end of the study. VH—vaginal hysterectomy, LSH—laparoscopic supracervical hysterectomy, SH—abdominal supracervical hysterectomy, TAH—abdominal total hysterectomy, TLH—laparoscopic total hysterectomy, BMI—body mass index.

**Table 2:** Urogenital Distress Inventory-6 (UDI-6), Incontinence Impact Questionnaire-7 (IIQ-7), The International Consultation on Incontinence Questionnaire (ICIQ) and the Female Sexual Function Index (FSFI) scores among the study group patients before and after hysterectomy.

Hysterectomy Type		UDI-6			IIQ-7			ICIQ			FSFI		
		Before	After	*p*	Before	After	*p*	Before	After	*p*	Before	After	*p*
VH (n = 103)	Mean ± SD	40.0 ± 28.3	33.1 ± 23.3	NS	30.0 ± 30.9	22.2 ± 26.4	NS	5.9 ± 5.9	4.8 ± 5.2	NS	23.0 ± 5.4	22.1 ± 10.0	NS
	Median	38.9	27.8		23.8	14.3		5	4				
LSH (n = 150)	Mean ± SD	25.9 ± 21.9	26.7 ± 19.0	NS	25.9 ± 21.9	14.6 ± 23.5	<0.001	4.4 ± 5.0	4.1 ± 4.5	NS	25.5 ± 5.4	26.5 ± 4.4	NS
	Median	16.7	19.4		16.7	0		4.0	3				
SH (n = 71)	Mean ± SD	25.3 ± 22.4	20.9 ± 18.4	NS	16.8 ± 25.7	11,6 ± 18.8	NS	1.2 ± 1.4	2.5 ± 3.7	*p* = 0.011	25.8 ± 6.1	25.8 ± 9.3	NS
	Median	22.2	16.7		0	0		1	0				
TAH (n = 47)	Mean ± SD	28.5 ± 21.7	28.9 ± 22.8	NS	16.3 ± 24.8	25.4 ± 22.9	NS	5.0 ± 6.0	5.1 ± 4.4	NS	22.8 ± 6.3	21.8 ± 4.1	NS
	Median	33.3	4		4.8	23.8		3.0	5				
TLH (n = 28)	Mean ± SD	18.4 ± 18.8	25 ± 15.5	NS	9.5 ± 18.7	17.1 ± 19.8	NS	2.5 ± 3.6	5.0 ± 4.4	NS	28.8 ± 5.1	20.2 ± 14.0	NS
	Median	13.9	22.2		0	14.3		0	4				
ALL (n = 399)	Mean ± SD	29.3 ± 24.4	27.1 ± 20.9	NS	23.2 ± 26.1	17.6 ± 23.6	<0.001	4.2 ± 5.1	4.1 ± 4.6	NS	24.9 ± 6.3	24.9 ± 6.3	NS
	Median	22.2	22.2		14.7	14.8		3	3.3				

VH—vaginal hysterectomy, LSH—laparoscopic supracervical hysterectomy, SH—abdominal supracervical hysterectomy, TAH—abdominal total hysterectomy, TLH—laparoscopic total hysterectomy.

**Table 3:** Urethral length among the study group patients before and after hysterectomy.

Hysterectomy Type		Urethral Length (cm)		
		Before	After	*p*
VH (n = 103)	Mean ± SD	2.9 ± 0.4	2.9 ± 0.3	NS
LSH (n = 150)	Mean ± SD	3.1 ± 0.5	3.0 ± 0.4	NS
SH(n = 71)	Mean ± SD	3.3 ± 0.4	3.2 ± 0.3	NS
TAH (n = 47)	Mean ± SD	3.1 ± 0.6	3.1 ± 0.4	NS
TLH (n = 28)	Mean ± SD	3.1 ± 0.5	3.0 ± 0.4	NS
ALL (n = 399)	Mean ± SD	3.1 ± 0.4	3.0 ± 0.3	NS

VH—vaginal hysterectomy, LSH—laparoscopic supracervical hysterectomy, SH—abdominal supracervical hysterectomy, TAH—abdominal total hysterectomy, TLH—laparoscopic total hysterectomy.

## Data Availability

Data is available with the author.
